# Apurinic/Apyrimidinic Endonucleases of *Mycobacterium tuberculosis* Protect against DNA Damage but Are Dispensable for the Growth of the Pathogen in Guinea Pigs

**DOI:** 10.1371/journal.pone.0092035

**Published:** 2014-05-06

**Authors:** Rupangi Verma Puri, P. Vineel Reddy, Anil K. Tyagi

**Affiliations:** Department of Biochemistry, University of Delhi South Campus, New Delhi, India; Texas A&M Health Science Center, United States of America

## Abstract

In host cells, *Mycobacterium tuberculosis* encounters an array of reactive molecules capable of damaging its genome. Non-bulky DNA lesions are the most common damages produced on the exposure of the pathogen to reactive species and base excision repair (BER) pathway is involved in the repair of such damage. During BER, apurinic/apyrimidinic (AP) endonuclease enzymes repair the abasic sites that are generated after spontaneous DNA base loss or by the action of DNA glycosylases, which if left unrepaired lead to inhibition of replication and transcription. However, the role of AP endonucleases in imparting protection against DNA damage and in the growth and pathogenesis of *M.tuberculosis* has not yet been elucidated. To demonstrate the biological significance of these enzymes in *M.tuberculosis*, it would be desirable to disrupt the relevant genes and evaluate the resulting mutants for their ability to grow in the host and cause disease. In this study, we have generated *M.tuberculosis* mutants of the base excision repair (BER) system, disrupted in either one (MtbΔ*end* or MtbΔ*xthA*) or both the AP endonucleases (MtbΔ*end*Δ*xthA*). We demonstrate that these genes are crucial for bacteria to withstand alkylation and oxidative stress *in vitro*. In addition, the mutant disrupted in both the AP endonucleases (MtbΔ*end*Δ*xthA*) exhibited a significant reduction in its ability to survive inside human macrophages. However, infection of guinea pigs with either MtbΔ*end* or MtbΔ*xthA* or MtbΔ*end*Δ*xthA* resulted in the similar bacillary load and pathological damage in the organs as observed in the case of infection with wild-type *M.tuberculosis*. The implications of these observations are discussed.

## Introduction

Despite the potentially intimidating environment of the macrophage, *M.tuberculosis* persists in the host as a result of several defense mechanisms [1], [2], [3], [4], [5], [6]. The repair of damaged DNA has been considered as an important mechanism for the survival of *M.tuberculosis* in the host [7]. The damages, that frequently occur to DNA as a consequence of reactive oxygen intermediates (ROI) and reactive nitrogen intermediates (RNI) produced by the macrophages, include the base modifications, generation of abasic sites and DNA strand breaks. The DNA damaged in such a manner represents the most common substrate for the Base excision repair (BER) pathway [8]. BER pathway is initiated by DNA glycosylases, a highly specialized class of enzymes, that specifically recognize and excise modified bases in DNA by hydrolyzing the N-glycosidic bond between the base and the sugar [9]. This step leads to the creation of abasic (also known as apurinic/apyrimidinic or AP) sites [9]. Abasic sites can also arise in DNA spontaneously [10]. The accumulation of AP sites in DNA is detrimental as they daunt essential processes such as replication and transcription [10]. For this reason, class II AP endonucleases are considered important enzymes that cleave the phosphodiester backbone on the 5′ end of the AP site leaving a 3′-hydroxyl group. DNA repair is completed by the actions of a DNA polymerase that fills in new base and DNA ligase that finally seals the gap [11], [12].

AP endonucleases have been classified into two families, the exonuclease III (ExoIII or Xth) and endonuclease IV (EndoIV or Nfo) families, based on their homology to the two *Escherichia coli* enzymes. In *E.coli*, XthA, which is the major AP endonuclease, represents 90% of the cellular AP endonuclease activity, while Nfo accounts for the remaining 10% activity [13]. In addition to AP endonuclease and 3′→5′ exonuclease activities, the *E.coli* AP endonucleases also exhibit additional 3′ phosphatase and 3′ phosphodiesterase activities that are responsible for removing a multitude of blocking groups, including 3′ phosphate and 3′ phosphoglycolate, that are present at single-stranded breaks in DNA, induced by oxidative agents [14], [15]. *Saccharomyces cerevisiae* also possesses two AP endonucleases, the Apn1 and Apn2 proteins that represent the EndoIV and the ExoIII family, respectively [16]. However, the major AP endonuclease in this organism is Apn1, that exhibits a strong AP endonuclease activity in yeast cells while the Apn2 protein, is a weak AP endonuclease that exhibits strong 3′→5′ exonuclease and 3′ phosphodiesterase activities [16], [17], [18], [19]. The human AP endonucleases, Ape1 and Ape2, are both members of the ExoIII family where Ape1 is the major human AP endonuclease. The EndoIV homologs are not known to be present in humans [20]. Although neither of the two AP endonuclease genes is universal, all species encode at least one of these genes, suggesting that AP endonuclease activity is required for all species [21].

*E.coli* mutant deficient in both the AP endonuclease genes (*nfo* and *xth*) have been demonstrated to display increased lethality to ionizing radiation and chemical oxidants implicating Nfo and Xth in the repair of such damages [22]. AP endonucleases have been evaluated for their importance in the pathogenesis of several bacteria such as *Salmonella typhimurium*, *Neisseria meningitides* and *Brucella abortus* [23], [24], [25]. To determine whether the repair of oxidatively damaged DNA is involved in the growth and pathogenesis of *S.typhimurium*, mutants of the AP endonuclease genes were generated that were deficient in one (*Δxth*, *Δnfo*) or both (*Δxth*/*nfo*) the AP endonucleases [23]. *S.typhimurium Δxth* and *Δxth*/*nfo* were significantly impaired for survival in RAW 264.7 murine macrophages and in C57BL/6 primary murine macrophages activated with IFN-γ [23]. In addition, *Δxth*/*nfo* was 12-fold attenuated when compared with the wild type in the murine typhoid fever model [23]. Two AP endonuclease paralogues namely NApe and NExo (both belonging to the Xth family) have been identified and characterized in the human pathogen *N.meningitides* [24]. By employing the mutants of these genes (Δ*nexo*, Δ*nape* and Δ*nexo:nape*), it has been demonstrated that these enzymes are necessary for the survival of *N.meningitidis* under oxidative stress [24]. In addition, the Δ*nexo* and Δ*nape* were recovered from the bloodstream of infected infant rats at significantly lower levels than the wild-type strain, the most significant reduction being observed in the case of double mutant (Δ*nexo:nape*) demonstrating that both NApe and NExo are required for the full virulence of *N.meningitides* [24]. The *Brucella abortus* possesses two *xthA* homologs (*xthA-1* and *xthA-2*) but have no homolog of *nfo* [25]. The *B.abortus xthA-1* mutant exhibited increased sensitivity to oxidative and alkylation stress when compared with the parental strain [25]. However, the mutant and the parental strains displayed equivalent spleen colonization profiles in BALB/c mice through 8 weeks post-infection and equivalent intracellular survival and replication profiles in the macrophages from C57BL/6 mice [25]. These authors suggested that residual AP endonuclease activity provided by XthA-2 may be responsible for the lack of attenuation of the *xthA-1* mutant in the murine model [25].

*M.tuberculosis*, is amongst the few bacteria to possess homologues of all known BER genes [21], [26]. The sequencing of *M.tuberculosis* genome revealed the presence of *E.coli* AP endonuclease homologs- XthA and Nfo namely, Exonuclease III (XthA) and Endonuclease IV (End) that are encoded by the genes *xthA* (Rv0427c) and *end* (Rv0670), respectively [27]. The biological importance of *xthA* in *M.tuberculosis* is highlighted by the fact that no variations in *xthA* have been observed in clinical strains [28]. Furthermore, a second AP endonuclease gene, encoding the endonuclease IV (End), is present in *M.tuberculosis* [27]. We have previously carried out biochemical characterization of the annotated *M.tuberculosis* AP endonucleases, namely Endonuclease IV (End) and Exonuclease III (XthA) and demonstrated that these proteins are functional AP endonucleases [29]. Given the importance of AP endonucleases in other bacteria, these proteins appear to be good targets to design anti-tubercular molecules. Besides, in a study by Sassetti and Rubin, these AP endonucleases have been implicated in the *in vivo* growth of the pathogen in mouse model of infection when the spleen colonization profiles of mice infected intravenously with an *M.tuberculosis* transposon mutant library were evaluated [30]. However, as suggested by the authors, this study had several limitations [30]. For example, the study employed a library of transposon mutants in *M.tuberculosis* to intravenously infect C57BL/6J mice, hence, the effect of single gene mutation in *M.tuberculosis* was not evaluated in isolation [30]. The phenotypes of individual AP endonuclease mutants observed in this study could have been influenced by the presence of fully virulent bacteria that predominated the pool which could have led to an accentuation of mutant phenotypes caused by competition. Besides, this study employed intravenous route of infection rather than the aerosol route which is the common route of infection for *M.tuberculosis*. Moreover, the animal model employed for these experiments may also have influenced the results of the identification of *in vivo* essential genes as the disease in mice differs markedly from human illness. Hence, though End and XthA seem to be important drug targets, these proteins need to be validated for their *in vivo* essentiality in a relevant model of experimental tuberculosis before their addition to the drug development pipeline. In the present study, we have deleted the AP endonuclease gene(s)–*end* or/and *xthA* from the *M.tuberculosis* genome to evaluate their biological significance to this pathogen and describe the phenotypes of these mutants in the context of their importance in defense against DNA damage, intracellular growth in macrophages and in the pathogenesis of *M.tuberculosis* by employing the guinea pig model of experimental tuberculosis.

## Materials and Methods

### Bacterial strains and growth conditions

The details of bacterial strains and plasmids used in this study are listed in Table 1. *Escherichia coli* strains XL-1 Blue (Stratagene, Heidelberg, Germany) and HB101 (Life Technologies, CA, USA) were used for cloning and were grown in Luria-Berteni (LB) broth or on LB agar. Mycobacterial strains were grown on Middlebrook (MB) 7H11 agar supplemented with 10% OADC (oleic-acid albumin dextrose catalase) and 0.2% glycerol or in MB7H9 broth supplemented with 10% ADC (albumin dextrose catalase), 0.2% glycerol and 0.05% Tween 80 at 37°C with shaking at 200 rpm. For the generation of mutants, *M.tuberculosis* H37Rv transformed with pJV53 was employed, as described previously [31]. Kanamycin and chloramphenicol were used at concentrations of 25 µg/ml and 30 µg/ml, respectively. Hygromycin was used at a concentration of 50 µg/ml for mycobacteria or at 150 µg/ml for *E.coli*.

**Table 1:** Bacterial strains, plasmids and cell line employed in this study.

Strains/Plasmids/Cell line	Description	Reference
**Strains**		
*E.coli* XL-1 Blue	*endA1 gyrA96*(*nalR*) *thi-1 recA1 relA1 lac glnV44* F′[::Tn*10 proAB*+ *lacI* ^q^ Δ(*lacZ*)M15] *hsdR17*(r_K_^−^ m_K_^+^)	Stratagene, Heidelberg, Germany
*E.coli* HB101	F–(*gpt*-*proA*) *62 leuB6 glnV44 ara-14 galK2 lacY1* (*mcrC-mrr*) *rpsL20* (Str^r^) *xyl-5 mtl-1 recA13*	Life Technologies, CA, USA
MtbWT *	*M.tuberculosis* expressing recombineering proteins gp60 and gp61/wild-type strain/parental strain	[36]
MtbΔ*end* *	*M.tuberculosis* H37Rv *end* mutant	This Study
MtbΔ*xthA* *	*M.tuberculosis* H37Rv *xthA* mutant	This Study
MtbΔ*end*Δ*xthA* *	*M.tuberculosis* H37Rv *end xthA* double mutant	This Study
MtbΔ*end*Comp*	MtbΔ*end* complemented with *end*	This Study
MtbΔ*xthA*Comp*	MtbΔ*xthA* complemented with *xthA*	This Study
**Plasmids**		
pYUB854	Cloning vector with hygromycin resistance gene cassette flanked with two multiple cloning sites	[57]
pJV53	Mycobacterium - *E.coli* shuttle vector encoding recombineering proteins gp60 and gp61	[31]
pVR1	A derivative of pSD5 containing chloramphenicol resistance gene under *M.tuberculosis* ribosomal RNA promoter	[32]
pYUBΔ*end*	pYUB854 with *end::hyg*	This Study
pYUBΔ*xthA*	pYUB854 with *xthA::hyg*	This Study
pYUBΔ*CATxthA*	pYUB854 with *xthA::CATtrrn*	This Study
plit38.proend	pLITMUS 38 carrying the *end* gene with native promoter	This Study
plit38.proxthA	pLITMUS 38 carrying the *xthA* gene with native promoter	This Study
pVR*end*	pVR carrying the *end* gene with native promoter	This Study
pVR*xthA*	pVR carrying the *xthA* gene with native promoter	This Study
**Cell line**		
THP-1	The human acute monocytic leukemia cell line	NCCS, Pune, India

* After generation of the mutants, all the strains were grown in the absence of kanamycin resulting in the loss of plasmid pJV53. The loss of plasmid pJV53 was confirmed by PCR analysis.

### Disruption of AP endonuclease genes and genetic complementation of the mutants

Oligonucleotides (Table 2) were designed to amplify (i) a ∼700 bp amplicon (amplicon I) comprising of ∼200 bp of the 5′ proximal region of the gene (*end* or *xthA*) in addition to a ∼500 bp region immediate upstream to it, and (ii) a ∼700 bp amplicon (amplicon II) comprising of ∼200 bp of the 3′ distal region of the gene (*end* or *xthA*) in addition to a ∼500 bp region immediate downstream to it. The amplicons I and II, specific for *end*, were PCR amplified by employing the oligonucleotides End-F1/End-R1 and End-F2/End-R2, respectively and cloned into the vector pYUB854 flanking the hygromycin cassette at *Xho*I/*Spe*I and *Kpn*I/*Xba*I, respectively, for the generation of pYUBΔ*end*. For the generation of pYUBΔ*xthA* the amplicons I and II, specific for *xthA*, were PCR amplified by employing the oligonucleotides XthA-F1/XthA-R1 and XthA-F2/XthA-R2, respectively and cloned into the vector pYUB854 flanking the hygromycin cassette at *Kpn*I/*Xba*I and *Xho*I/*Spe*I, respectively. The linear allelic exchange substrates (AES), *end::hyg* and *xthA::hyg* were excised from pYUBΔ*end* and pYUBΔ*xthA*, respectively, by using the restriction enzymes *Kpn*I*/Spe*I and electroporated into wild-type *M.tuberculosis* carrying pJV53 separately, to generate the *end* (MtbΔ*end*) and *xthA* (MtbΔ*xthA*) mutants of *M.tuberculosis* [31]. For the generation of a double mutant of *M.tuberculosis*, *xthA* was disrupted in MtbΔ*end*. Briefly, a hygromycin resistance cassette in pYUBΔ*xthA* was removed by using *Xba*I and *Xho*I and was replaced with the chloramphenicol resistance gene expressed under the *M.tuberculosis* ribosomal RNA promoter to generate pYUBΔ*CATxthA*. This vector was digested with *Kpn*I/*Spe*I and the resulting AES (Δ*xthA::CATtrrn*) was electroporated into MtbΔ*end* to generate the double mutant of *M.tuberculosis* (MtbΔ*end*Δ*xthA*). For the generation of mutants by employing recombineering method, wild-type *M.tuberculosis* carrying pJV53 (for the generation of MtbΔ*end* and MtbΔ*xthA*) and MtbΔ*end* (for the generation of MtbΔ*end*Δ*xthA*) were cultured separately in MB7H9, 4% OADC, 0.05% Tween 80 and 25 µg/ml kanamycin at 37°C with shaking at 200 rpm, sub-cultured in MB7H9, 0.05% Tween 80, 25 µg/ml kanamycin and 0.2% succinate, grown to mid-log phase and induced by the addition of 0.2% acetamide. After growth for 48 h at 37°C, electrocompetent cells were prepared and transformed with AES.

**Table 2:** Oligonucleotides employed in this study.

Oligonucleotides	Sequence (5′→3′)
End-F1	aattatactagtgctccgagtaggtatcgccatac
End-R1	aattatctcgagggtagggcgcatgcacgtagatg
End-F2	gatatctctagatcgatctggtgcactgcaacgac
End-R2	gatatcggtaccgtaggacatgatggcaccgttgc
End-F3	gatatcagatctccgtgatcagtgtgttctgc
End-R3	gatatcagatcttcagctgccggttctttcccg
End-F	gttgccggccgaaagcc
End-R	tgtggatgaccgacaccg
XthA-F1	gataccggtaccagcgccgagagcaagtgttgg
XthA-R1	gatacctctagaaagccaatcgaggacacgatccaacc
XthA-F2	aattatctcgagggtttacacctactgggattac
XthA-R2	aattatactagtttgagcttccgcaatagcaacgc
XthA-F3	gagtgtgaatcctgcgacatgcccgacggcacaattgac
XthA-R3	gatatcggatccctacccggcgtgcaggtcgacgag
XthA-F4	gatatcggatccgcaaccgctttaccagcatcgggtc
XthA-R4	gtcaattgtgccgtcgggcatgtcgcaggattcacactc
XthA-F	accatcacagaaatcagcg
XthA-R	cttgctcgctttggctggcg

To facilitate genetic complementation, a 1233 bp PCR product (proend) encompassing *end* gene (759 bp) along with its promoter region (474 bp) was PCR amplified by using *M.tuberculosis* H37Rv genomic DNA as template by employing the oligonucleotides End-F3/End-R3. The above PCR product was cloned at *Eco*RV site in pLITMUS38 plasmid and the clone was confirmed by DNA sequencing. proend was excised out from plit38.proend by using *Bgl*II, end-repaired by Klenow polymerase and cloned in pVR1 plasmid (digested by using *Mlu*I/*Xba*I and end-repaired by Klenow polymerase) [32]. The resulting plasmid pVR*end*, was electroporated into MtbΔ*end* to generate the complemented strain, MtbΔ*end*Comp. For the complementation of MtbΔ*xthA* mutant, two separate PCR products were amplified- a 876 bp region of *xthA* gene was PCR amplified by employing oligonucleotides XthA-F3/XthA-R3 and a 441 bp promoter region of *xthA* (immediately upstream to Rv0429c) was PCR amplified by employing oligonucleotides XthA-F4/XthA-R4. By employing these two PCR products as template, PCR was performed by employing the oligonucleotides XthA-F4/XthA-R3 by the overlap extension method as described previously [33]. The resulting PCR product, proxthA (1317 bp) was digested with *Eco*RV and cloned at *Eco*RV site in pLITMUS38 plasmid and the clone was confirmed by DNA sequencing. proxthA was excised out from plit38.proxthA by using *Eco*RV and cloned in pVR1 plasmid (digested by using *Mlu*I/*Xba*I and end-repaired by Klenow polymerase). The resulting plasmid pVR*xthA*, was electroporated into MtbΔ*xthA* to generate the complemented strain, MtbΔ*xthA*Comp.

### Sensitivity to DNA damaging agents

To evaluate the ability of the AP endonuclease mutants to withstand DNA damage, the wild-type and the mutant strains were subjected to different concentrations of DNA damaging agents, such as methyl methane sulfonate (MMS), hydrogen peroxide (H_2_O_2_) and mitomycin C (MMC) for 24 h and the effect of the deletion of the gene(s) on the survival of *M.tuberculosis* was evaluated by CFU enumeration. Briefly, early log phase cultures of MtbWT, MtbΔ*end*, MtbΔ*xthA* and MtbΔ*end*Δ*xthA* were diluted to an A_600 nm_ of 0.1. The cultures were pelleted and the cells were washed once with equal volume of PBS (pH 7.4) and resuspended in MB7H9 media (without OADC supplementation) containing the desired concentration of stress agent. Each *M.tuberculosis* strain was also resuspended in MB7H9 media alone (without any stress agent) which was used as a control for that strain. The cultures were incubated at 37°C for 24 h. Based on the earlier studies on these DNA damaging agents in other bacterial systems such as *E.coli* and *S.typhimurium* [13], [34], [35], the initial standardization experiments were carried out in *M.tuberculosis* by employing a broad range of concentration in order to select the final concentration range for the studies. After a 24 h exposure, cells were diluted and plated on MB7H11 supplemented with OADC. Colonies were counted after 3 weeks of incubation at 37°C. The survival of *M.tuberculosis* strains was expressed as percent survival by using the formula: Percent survival = 100×(Number of bacteria obtained post stress/Number of bacteria obtained without addition of stress agent). Experiment was performed in duplicates and was repeated twice.

### Intracellular growth in human THP-1 macrophages

Human monocytic THP-1 cells were cultured in RPMI-GlutaMAX medium [containing 10% heat inactivated fetal bovine serum (FBS) and 1% antibiotic-antimycotic mix] (Gibco Invitrogen Life Technologies, NY, USA) at 37°C in 5% CO_2_ and infected as described previously [36]. Briefly, THP-1 macrophages were seeded at 5×10^5^ cells per well in 24-well tissue culture plates and differentiated to macrophages by using 30 nM phorbol 12-myristate 13-acetate (PMA) (Sigma, MO, USA) for 16 h at 37°C in 5% CO_2_. Cells were washed with RPMI medium and rested for 2 h before infection in 1 ml RPMI medium supplemented with 10% FBS. The monolayers were infected with wild-type *M.tuberculosis*, MtbΔ*end*, MtbΔ*xthA* and MtbΔ*end*Δ*xthA* separately, at a multiplicity of infection of 1 bacterium per 5 THP-1 macrophages for 4 h at 37°C in triplicates, following which the monolayers were washed twice with the medium. Subsequently, extracellular bacteria were removed by treatment with 200 µg/ml amikacin for 2 h at 37°C. On days 0 (6 h), 2, 4 and 6, the infected macrophage monolayers (three wells per strain) were lysed with 1 ml of 0.025% SDS (Sigma, MO, USA) to release intracellular mycobacteria, which were then enumerated by plating serial dilutions on MB7H11 agar. Colonies were counted after 4 weeks of incubation at 37°C and the data were expressed as CFU/ml. The extent of THP-1 infection by *M.tuberculosis* strains was calculated by employing the formula−100×(Number of intracellular bacteria obtained on Day 0 (i.e. 6 h post infection) per well/Number of THP-1 macrophages seeded per well).

### *In vivo* guinea pig experiments

Pathogen-free out-bred female guinea pigs of the Duncan-Hartley strain in the weight range of 250 to 350 g were obtained from the Disease Free Small Animal House Facility, Chaudhary Charan Singh Haryana Agricultural University, Hisar, India. The animals were maintained in a biosafety level 3 facility and routinely cared for according to the guidelines of the CPCSEA (Committee for the Purpose of Control and Supervision on Experiments on Animals), India. To study the influence of *end* or/and *xthA* disruption on the growth and pathogenesis of *M.tuberculosis*, guinea pigs were infected by the aerosol route with 10 to 30 bacilli of either wild-type *M.tuberculosis*, MtbΔ*end*, MtbΔ*end*Comp, MtbΔ*xthA*, MtbΔ*xthA*Comp or MtbΔ*end*Δ*xthA*. Animals (n = 5) were euthanized at 4 weeks and 10 weeks post-infection by CO_2_ asphyxiation. After dissecting the animals, lungs, liver and spleen were scored for gross pathological damage such as the extent of involvement of the organ, number and size of tubercles, areas of inflammation and damage due to necrosis. The gross pathological scores were graded from 1–4 based on the modified Mitchison scoring system [37], [38]. Left caudal lung lobe and caudal segment of spleen from the infected animals were aseptically removed for bacterial enumeration. The specific segments of lung and spleen were weighed and homogenized separately in 5 ml saline by using a polytron homogenizer. Appropriate dilutions of the homogenates were plated on MB7H11 agar plates in duplicates and incubated at 37°C for 3–4 weeks. Colonies were counted and expressed as mean log_10_ CFU/organ. For histopathological evaluation, the right lung and a portion of left dorsal lobe of liver from the infected animals were removed and fixed in 10% buffered formalin. 5 µm thick sections from the formalin fixed, paraffin embedded tissues were stained with haematoxylin and eosin (H&E). The tissues were coded and the coded samples were analysed by a certified pathologist having no knowledge of the experimental groups.

### Ethics statement

Protocols for all the animal experiments included in this manuscript along with the requirement of guinea pigs were reviewed and approved by the Institutional Animal Ethics Committee of University of Delhi South Campus, New Delhi, India (Ref. No. 1/IAEC/AKT/BIOCHEM/UDSC/14.10.2011). All animals were routinely cared for according to the guidelines of the CPCSEA (Committee for the Purpose of Control and Supervision of Experiments on Animals). The guinea pigs were euthanized by CO_2_ asphyxiation and all efforts were made to minimize animal suffering.

### Statistical analysis

For comparing the i) sensitivity of *M.tuberculosis* strains against DNA damaging agents and ii) growth of mycobacterial strains in THP-1 cells, two-way analysis of variance (ANOVA) with the Bonferroni multiple comparison test was employed. For the comparison of bacillary load in the lungs or spleen of infected guinea pigs, one-way ANOVA with the Tukey post test was employed. For comparison of the gross pathological scores of various groups, the nonparametric Kruskal-Wallis test followed by the Mann-Whitney U test was employed. Differences were considered significant when *P*<0.05. For the statistical analysis and generation of graphs, Prism 5 software (version 5.01; GraphPad Software Inc., CA) was used.

## Results

### Disruption of the AP endonuclease gene(s) in *M.tuberculosis* and characterization of the mutants

**Figure 1:**
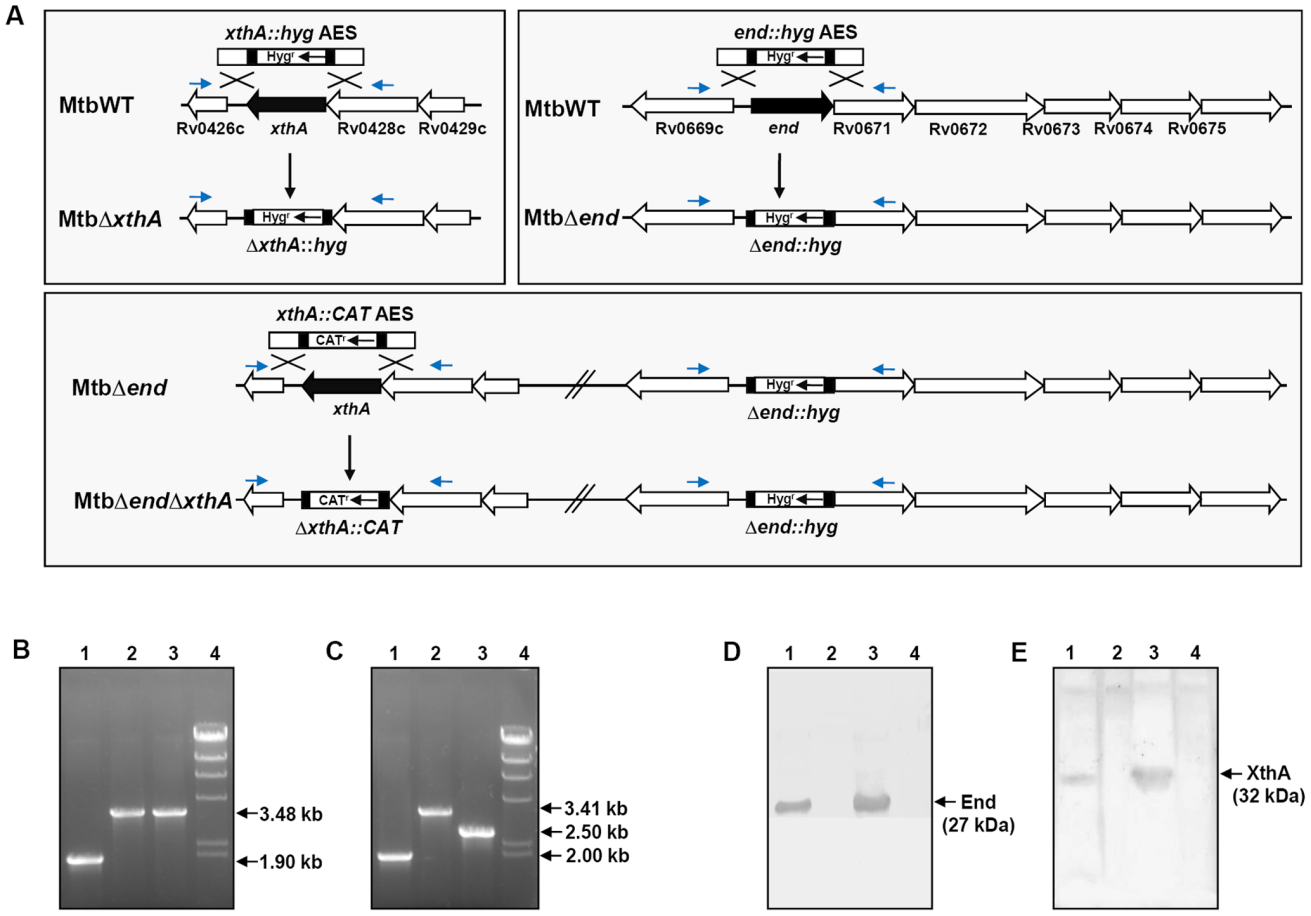
Characterization of AP endonuclease mutants of *M.tuberculosis*. (A) Strategy for disruption of *xthA* or/and *end* genes in wild-type *M.tuberculosis*. The figure depicts recombineering in wild-type *M.tuberculosis* between *xthA::hyg* AES and *xthA* gene to generate MtbΔ*xthA* and between *end::hyg* AES and *end* gene to generate MtbΔ*end*. Recombineering in MtbΔ*end* between *xthA::CAT* AES and *xthA* gene was employed to generate MtbΔ*end*Δ*xthA*. The location of the primers employed for the confirmation of gene deletion by PCR is indicated by small arrows. (B) Confirmation of deletion of *end* by PCR. PCR resulted in a 1.9 kb amplicon for wild-type *M.tuberculosis* (lane 1) and 3.48 kb amplicon for MtbΔ*end* as well as MtbΔ*end*Δ*xthA* (lane 2 and lane 3, respectively). Lane 4 - λ*Hin*dIII ladder. (C) Confirmation of deletion of *xthA* by PCR. PCR resulted in a 2.0 kb amplicon for wild-type *M.tuberculosis* (lane 1), 3.41 kb amplicon for MtbΔ*xthA* (lane 2) and 2.5 kb amplicon for MtbΔ*end*Δ*xthA* (lane 3). Lane 4 - λ*Hin*dIII ladder. (D) Confirmation of deletion of *end* by immunoblot analysis. 10 µg (estimated by Bradford protein assay) lysates of wild-type *M.tuberculosis* (lane 1), MtbΔ*end* (lane 2), MtbΔ*end*Comp (lane 3) and MtbΔ*end*Δ*xthA* (lane 4) were separated on a 12% polyacrylamide gel. Anti-End polyclonal antibody was employed to detect End (a 27 kDa band) in the lysates of wild-type *M.tuberculosis* (lane 1) and MtbΔ*end*Comp (lane 3) while the disruption of *end* in MtbΔ*end* (lane 2) and MtbΔ*end*Δ*xthA* (lane 4) was confirmed by the absence of this band. (E) Confirmation of deletion of *xthA* by immunoblot analysis. 30 µg (estimated by Bradford protein assay) lysates of wild-type *M.tuberculosis* (lane 1), MtbΔ*xthA* (lane 2), MtbΔ*xthA*Comp (lane 3) and MtbΔ*end*Δ*xthA* (lane 4) were separated on a 12% polyacrylamide gel. Anti-XthA polyclonal antibody was employed to detect XthA (as a 32 kDa band) in the lysates of wild-type *M.tuberculosis* (lane 1) and MtbΔ*xthA*Comp (lane 3) while the disruption of *xthA* in MtbΔ*xthA* (lane 2) and MtbΔ*end*Δ*xthA* (lane 4) was confirmed by the absence of this band. (Ponceau S Staining Solution was used to verify equivalent transfer of proteins from the gels to the polyvinylidene difluoride membranes during immunoblotting in D and E).

To investigate the importance of AP endonucleases in *M.tuberculosis*, we employed recombineering method to generate three deletion mutants of *M.tuberculosis* lacking *end* (MtbΔ*end*), *xthA* (MtbΔ *xthA*) and both the genes (MtbΔ*endΔxthA*) (Fig. 1A) [31]. The frequency of mutant isolation was observed to range between 63% and 88% and was calculated by using the formula−100×(Number of legitimate recombinants confirmed by PCR)/(Number of transformants screened). The mutations were confirmed by three approaches. (i) We confirmed all the deletion mutations by PCR analysis. Primers employed for the PCR confirmation were designed 100 bp external to the linear AES and are indicated as blue arrows in figure 1A (Table 2). To confirm the disruption of *end*, we carried out PCR amplification by employing the oligonucleotides End-F and End-R (Fig. 1B). DNA from wild-type *M.tuberculosis* yielded a PCR product of 1.90 kb while the DNA from MtbΔ*end* or MtbΔ*end*Δ*xthA* resulted in a PCR product of 3.48 kb corresponding to replacement of 381 bp region in *end* by the hygromycin resistance cassette (1.95 kb) (Fig. 1B). Similarly, to confirm the disruption of *xthA*, PCR amplifications were carried out by employing the oligonucleotides XthA-F and XthA-R (Fig. 1C). DNA from wild-type *M.tuberculosis* yielded a PCR product of 2.0 kb. The DNA from MtbΔ*xthA* yielded a PCR product of 3.41 kb corresponding to replacement of 554 bp region in *xthA* by the hygromycin resistance cassette. The DNA from MtbΔ*end*Δ*xthA* resulted in a PCR product of 2.50 kb, corresponding to replacement of 554 bp region of *xthA* by chloramphenicol resistance gene expressed under the *M.tuberculosis* ribosomal RNA promoter (1.05 kb) (Fig. 1C). (ii) The above amplification products were subjected to DNA sequencing that further confirmed the disruption of *end* in MtbΔ*end*, *xthA* in MtbΔ*xthA* and both the genes in MtbΔ*end*Δ*xthA*. (iii) The disruption of AP endonuclease genes was further confirmed by immunoblot analysis by employing polyclonal antibodies raised against the respective proteins (Fig. 1D and 1E). In the case of cell free protein extracts from wild-type *M.tuberculosis*, the presence of 27 kDa and 32 kDa bands confirmed the presence of End (Fig. 1D) and XthA (Fig. 1E), respectively. These End and XthA bands (27 kDa and 32 kDa, respectively) were absent in the immunoblot analysis carried out by employing the cell free protein extracts from the MtbΔ*end* and MtbΔ*xthA*, respectively, while either protein was absent in the cell free protein extract of MtbΔ*end*Δ*xthA*. Complementation of *end* in the case of MtbΔ*end*Comp or *xthA* in the case of MtbΔ*xthA*Comp was confirmed by the restoration of 27 kDa End band or 32 kDa XthA band, respectively, when the cell free protein extracts from these strains were subjected to immunoblot analysis. When the growth characteristics of *M.tuberculosis* strains were assessed under standard culture conditions in MB7H9 media, the AP endonuclease mutants displayed no significant difference in growth when compared with wild-type *M.tuberculosis* (data not shown). Thus, *end* and *xthA* are dispensable for the *in vitro* growth of *M.tuberculosis*.

### Influence of the disruption of AP endonuclease genes on the susceptibility of *M.tuberculosis* to DNA damaging agents

To evaluate the role of End and XthA in mediating the response of *M.tuberculosis* to DNA damage, MtbΔ*end*, MtbΔ*xthA*, MtbΔ*end*Δ*xthA* and MtbWT were subjected to various DNA damaging agents, such as methyl methane sulfonate (MMS, an alkylating agent), hydrogen peroxide (H_2_O_2_, an inorganic peroxide) and mitomycin C (MMC, a DNA cross linker) as described in the materials and methods. The initial standardization experiments for stress carried out in broth, employed a concentration range of 0.2 to 10 mM MMS, 0.2 to 10 mM H_2_O_2_ and 0.1 to 10 µM MMC against wild-type *M.tuberculosis*. Based on the results from these studies, a concentration range of 0.5 to 2.0 mM in the case of MMS, 0.5 to 2.0 mM in the case of H_2_O_2_ and 1 to 10 µM in the case of MMC was employed for the assay.

**Figure 2:**
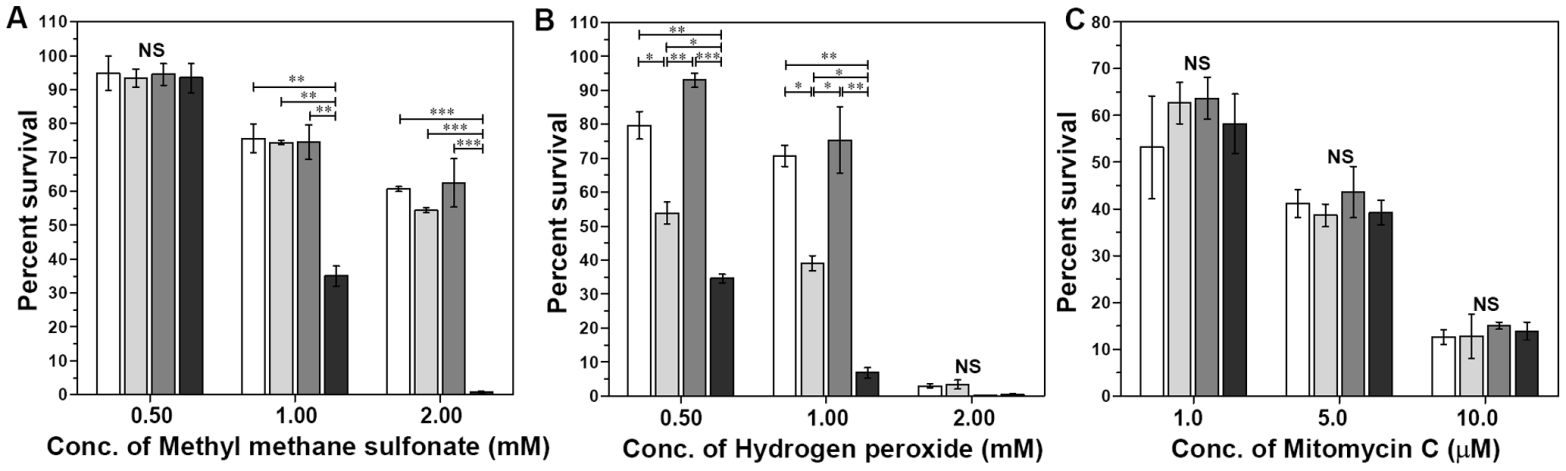
Influence of disruption of AP endonuclease(s) on the susceptibility of *M.tuberculosis* to DNA damaging agents. The figure represents the fraction of surviving bacteria after a 24(A) methyl methane sulfonate (MMS), (B) hydrogen peroxide (H_2_O_2_) and (C) mitomycin C (MMC). The results indicate that while both the AP endonucleases are important in protecting the pathogen against alkylation damage, End plays a more important role than XthA in protecting this pathogen against oxidative assault. End and XthA do not contribute to the repair of intrastrand and interstrand cross-linking of DNA. The percentage of the bacteria surviving after addition of the stress agent in comparison to the bacteria without the addition of stress agent is represented as the mean (±SE) of two independent experiments carried out in duplicates. NS, not significant; *, *P*<0.05; **, *P*<0.001 and ***, *P*<0.001 (One way ANOVA).

The influence of disruption of AP endonuclease(s) on the ability of *M.tuberculosis* to withstand alkylation stress was measured by exposure to methymethane sulfonate (MMS) (Figure 2A). At a concentration of 0.5 mM, MMS displayed no effect on the survival of any of the *M.tuberculosis* strains. However, when the concentration of MMS was further increased, the differences between the single gene mutants and double gene mutant became apparent. At a concentration of 1 mM MMS, the survival of MtbWT (75.63%), MtbΔ*end* (74.39%) and MtbΔ*xthA* (74.49%) strains was comparable, suggesting that the function of any of these two enzymes can be compensated by the other under these experimental conditions. However, a clear indication of the importance of these enzymes became apparent when the sensitivity of MtbΔ*end*Δ*xthA* towards MMS was evaluated. The double gene mutant exhibited a significantly enhanced sensitivity to the toxic effect of MMS at a concentration of 1 mM as demonstrated by a significantly reduced survival (35%), when compared with the wild-type strain, indicating thereby that these AP endonucleases play important role in protecting the pathogen against the alkylation damage (Figure 2A). Similarly, at a concentration of 2 mM MMS, while the survival of the parental (60.63%), MtbΔ*end* (54.39%) and MtbΔ*xthA* (62.49%) strains was comparable, the survival of MtbΔ*end*Δ*xthA* significantly reduced by 100 folds (0.63%) when compared with the parental strain (Figure 2A). These results signify an important role of AP endonucleases in withstanding alkylation stress in *M.tuberculosis*.

The influence of disruption of AP endonuclease(s) on the ability of *M.tuberculosis* to withstand oxidative stress was measured by exposure to hydrogen peroxide (H_2_O_2_) (Figure 2B). At a concentration of 0.5 mM H_2_O_2_, the survival of MtbWT and MtbΔ*xthA* was 79.64% and 92.91%, respectively, which was not significantly different from each other. However, the survival of MtbΔ*end* (53.84%) was significantly reduced in comparison to the parental strain. These observations indicate that at this concentration of H_2_O_2_, End protects bacteria from oxidative stress whereas XthA does not. However, at this concentration of H_2_O_2_, the highest sensitivity to peroxide damage was exhibited by the double gene mutant i.e. MtbΔ*end*Δ*xthA* which exhibited a significant reduction in its survival (34.57%) in comparison to the parental strain indicating the importance of AP endonucleases in protecting the pathogen against oxidative damage (Figure 2B). Similarly, at a concentration of 1 mM H_2_O_2_, it was observed that the survival of MtbWT (70.66%) and MtbΔ*xthA* (75.27%) was not significantly different from each other, while the survival of MtbΔ*end* (39.08%) was significantly reduced in comparison to the wild-type strain. The double mutant was maximally sensitive at this concentration with a significantly reduced survival of 6.85% in comparison to the wild-type strain. At a concentration of 2 mM H_2_O_2_, all the strains exhibited a markedly reduced survival with no statistical differences in their sensitivity to peroxide stress. Taken together, these observations point out that while both the AP endonucleases are important in protecting *M.tuberculosis* from the damage caused by oxidative radicals, their contribution may not necessarily be comparable and End appears to be playing a more important role than XthA because of its higher capacity to withstand oxidative damage.

The AP endonuclease mutants were also examined for their sensitivity against another DNA damaging agent, mitomycin C (MMC) along with the wild-type strain (Figure 2C). The survival of MtbΔ*end*, MtbΔ*xthA* and MtbΔ*end*Δ*xthA* was similar when compared with MtbWT at all the concentrations of the stress agent employed in the assay (Figure 2C). The similar sensitivity of MtbWT, MtbΔ*end*, MtbΔ*xthA* and MtbΔ*end*Δ*xthA* against MMC suggest that End and XthA do not contribute to the repair of DNA damages produced in response to the DNA cross linker MMC.

### MtbΔ*end*Δ*xthA* exhibits attenuated growth in human THP-1 macrophages

**Figure 3:**
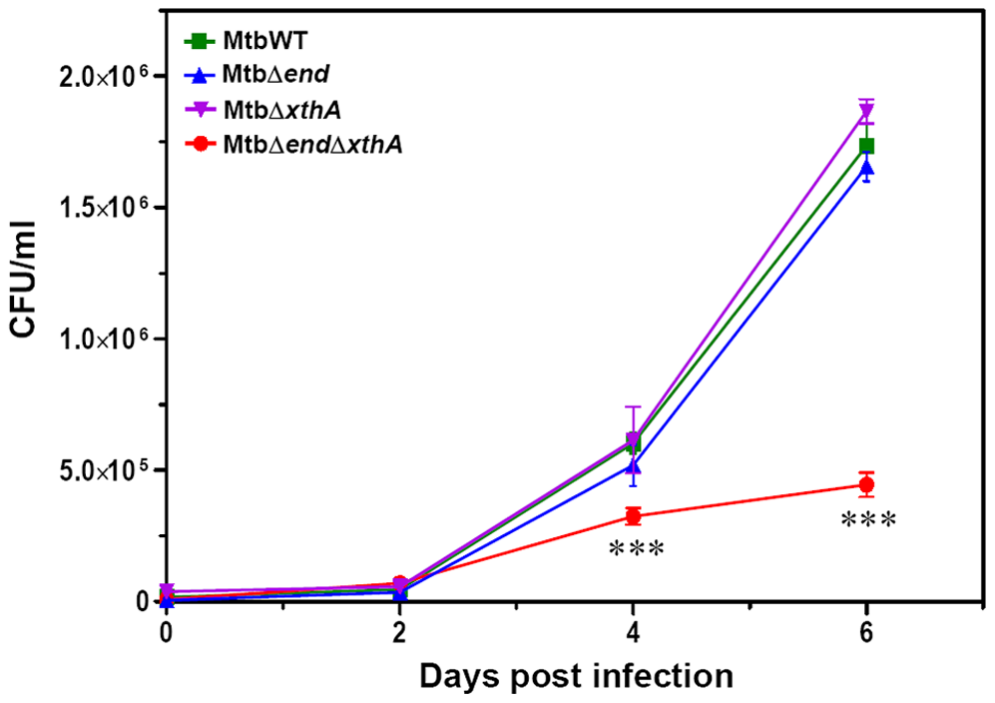
Disruption of both the AP endonucleases impairs the growth of *M.tuberculosis* in THP-1 macrophages. THP-1 cells were infected with wild-type *M.tuberculosis*, MtbΔ*end*, MtbΔ*xthA* or MtbΔ*end*Δ*xthA* at an MOI of 1∶5 (bacteria∶macrophage). The number of intracellular viable bacteria was determined on each alternative day for 6 days. A significant attenuation in the growth of MtbΔ*end*Δ*xthA* in comparison to wild-type *M.tuberculosis* was observed on the 4^th^ and 6^th^ day of infection. The growth of single mutants MtbΔ*end* and MtbΔ*xthA* was comparable with wild-type *M.tuberculosis*. The values are represented as the mean (±SE) of three independent infections and the experiment was repeated three times. ***, *P*<0.001 (Two way ANOVA).

Intracellular growth of MtbWT, MtbΔ*end*, MtbΔ*xthA* and MtbΔ*end*Δ*xthA* was compared in the THP-1 human macrophage cell line. The extent of THP-1 infection by *M.tuberculosis* strains by employing a multiplicity of infection of 1 bacterium per 5 macrophages was 15–20%. Wild-type *M.tuberculosis* grew normally in THP-1 macrophages upto 6 days post-infection. During this time period, the growth of MtbWT, MtbΔ*end* and MtbΔ*xthA* was comparable. Initially, upto 2 days post-infection, no significant difference was observed in the growth of double gene mutant (MtbΔ*end*Δ*xthA*) when compared with the wild-type strain. However, thereafter, MtbΔ*end*Δ*xthA* exhibited a significant attenuation in its growth with a 2 fold and 4 fold reduction in CFU at 4 and 6 days post-infection, respectively, in comparison to wild-type *M.tuberculosis* (Fig. 3). These observations with the double gene mutant show that End and XthA are important for intracellular survival of *M.tuberculosis*. However, the fact that MtbΔ*end*, MtbΔ*xthA* and MtbWT exhibited a comparable intracellular growth suggests that the loss of a singular activity i.e. either End or XthA can be compensated by the presence of the other enzyme.

### Disruption of AP endonuclease(s) has no affect on the growth of *M.tuberculosis* in guinea pig model of infection

**Figure 4:**
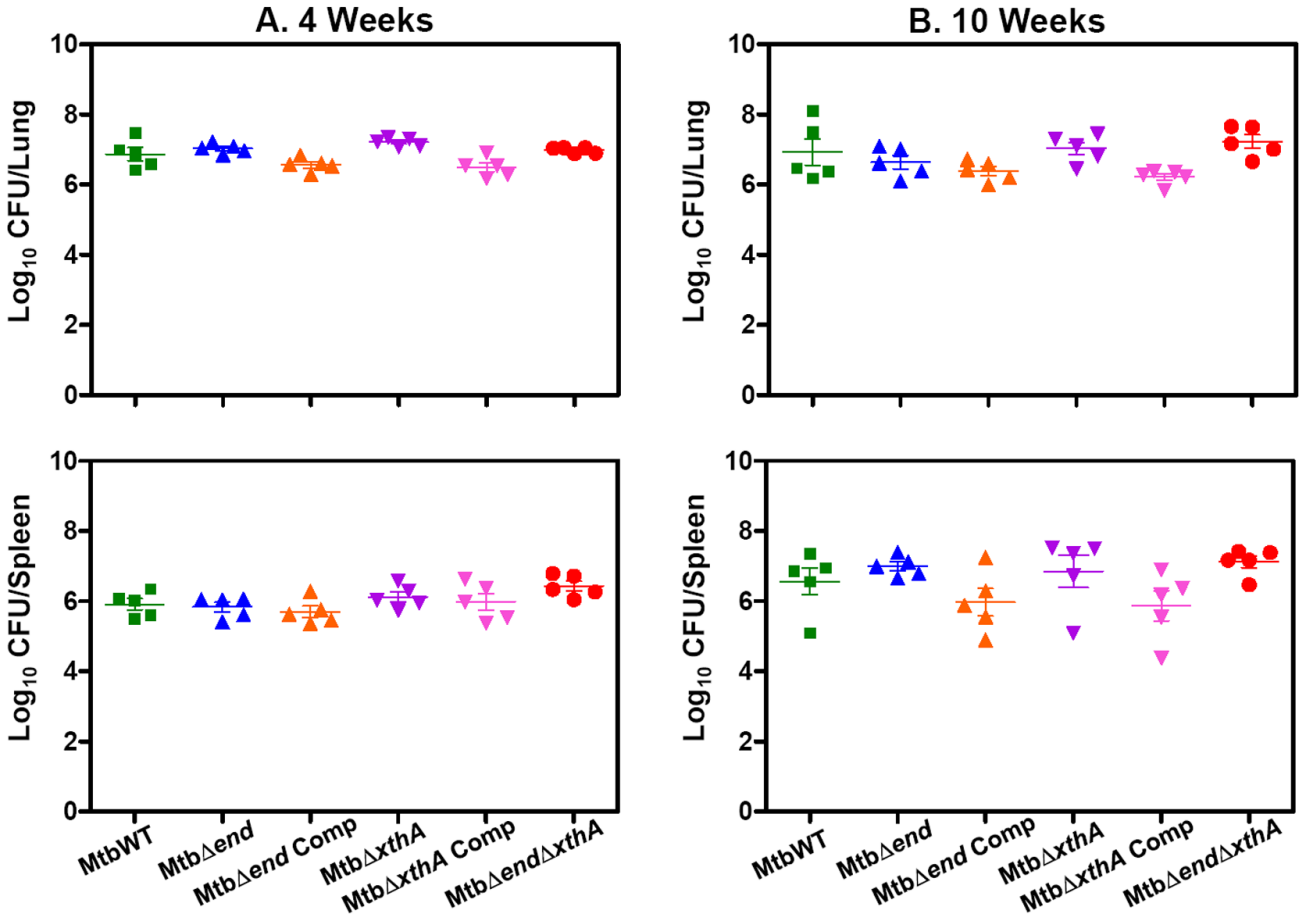
Influence of disruption of AP endonuclease(s) on the growth of *M.tuberculosis* in guinea pigs. The figure depicts bacillary load in the lungs and spleens of guinea pigs (n = 5) infected with wild-type *M.tuberculosis*, MtbΔ*end*, MtbΔ*end*Comp, MtbΔ*xthA* and MtbΔ*xthA*Comp and MtbΔ*end*Δ*xthA* at (A) 4 weeks and (B) 10 weeks post-infection. Guinea pigs belonging to all groups exhibited a comparable bacillary load in the lungs as well as in the spleen. Each data point represents the Log_10_ CFU value for an individual animal and the bar depicts mean (±SE) for each group.

In order to elucidate the importance of AP endonucleases in the growth of *M.tuberculosis* in the host, we have employed guinea pig model of experimental tuberculosis. Guinea pigs were infected with 10–30 bacilli of MtbWT, MtbΔ*end*, MtbΔ*end*Comp, MtbΔ*xthA*, MtbΔ*xthA*Comp or MtbΔ*end*Δ*xthA* by using aerosol route of infection and euthanized at 4 weeks (Fig. 4A) and 10 weeks (Fig. 4B) post-infection. At 4 weeks post-infection, the bacillary load in the lungs of the guinea pigs infected with any of the *M.tuberculosis* strains was comparable (6.5 to 7.2 log_10_ CFU). Similarly, the spleens of the guinea pigs infected with various *M.tuberculosis* strains, exhibited a comparable bacillary load (5.7 to 6.4 log_10_ CFU) (Fig. 4A). Similarly, at 10 weeks post-infection also, the bacillary load in the lungs of animals infected with any of the *M.tuberculosis* strains was comparable and corresponded to a range of 6.2 to 7.2 log_10_ CFU, while the bacillary load in the spleens of these animals corresponded to a range of 5.8 to 7.1 log_10_ CFU (Fig. 4B). This data indicates that the absence of AP endonucleases does not influence the growth of *M.tuberculosis* in the guinea pig infection model.

### Infection of guinea pigs with *M.tuberculosis* or AP endonuclease mutants results in comparable pathological damage

The gross pathological damage in the organs of the guinea pigs infected with MtbWT, MtbΔ*end*, MtbΔ*end*Comp, MtbΔ*xthA*, MtbΔ*xthA*Comp or MtbΔ*end*Δ*xthA*, was comparable at 4 weeks post-infection. The lungs of the guinea pigs infected with any of the *M.tuberculosis* strains displayed moderate involvement with occasional large tubercles. Hepatic and splenic tissues of guinea pigs infected with the parental, mutant or the complemented strains exhibited numerous small sized tubercles. No significant differences were observed in the gross pathological scores for lungs, liver or spleen of the animals infected with the parental, mutant or the complemented strains (data not shown). Moreover, commensurate to the gross pathological findings, the lung and liver of the animals infected with MtbWT, MtbΔ*end*, MtbΔ*end*Comp, MtbΔ*xthA*, MtbΔ*xthA*Comp or MtbΔ*end*Δ*xthA* exhibited a comparable histopathological damage. The lungs of infected animals exhibited granulomatous inflammation encompassing ∼30% of the total lung area while the livers displayed minimal involvement comprising ∼5% of liver area (data not shown).

**Figure 5:**
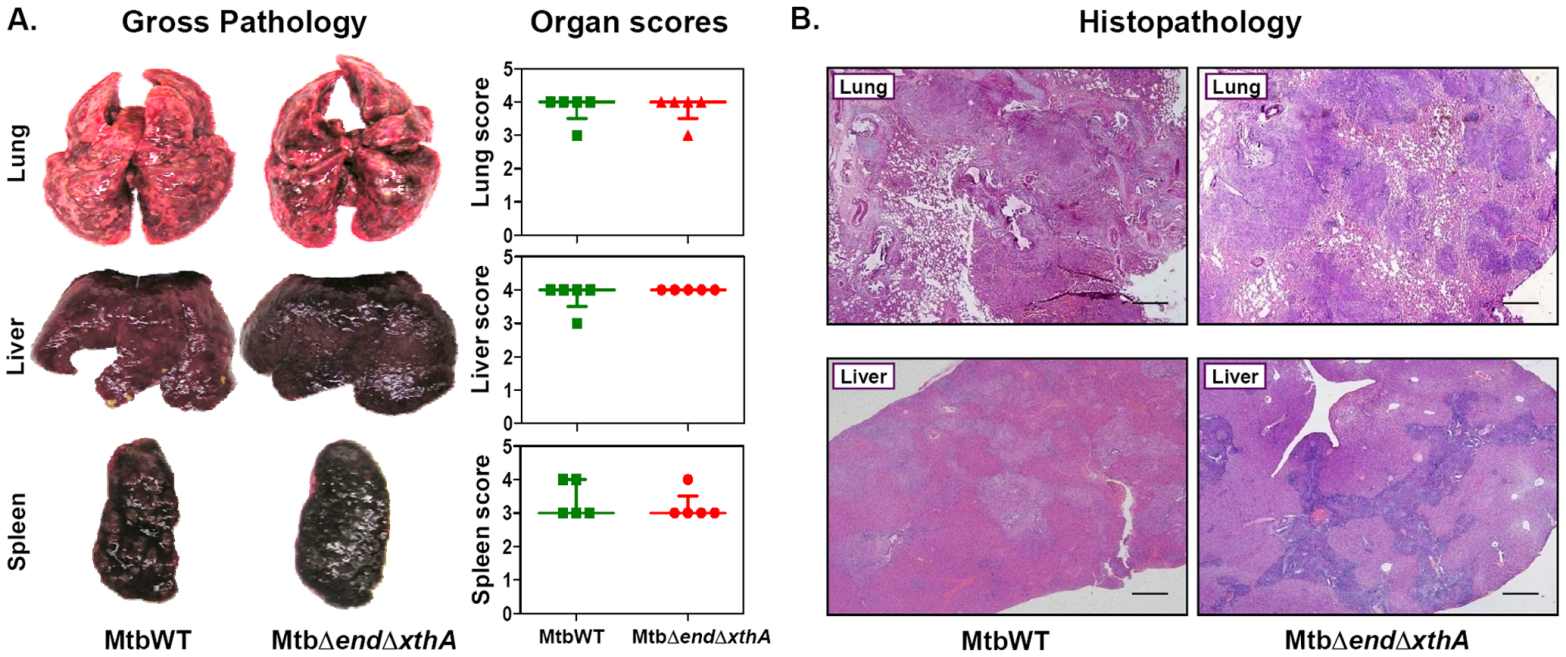
Gross pathological and histopathological damage in the organs of infected guinea pigs. (A) The figure depicts representative photographs of gross pathological lesions and graphical depiction of gross scores of lungs, liver and spleen of guinea pigs (n = 5) infected with wild-type *M.tuberculosis* or MtbΔ*end*Δ*xthA* euthanized at 10 weeks post-infection. The organs of guinea pigs infected with either wild-type *M.tuberculosis* or MtbΔ*end*Δ*xthA* exhibited heavy involvement with numerous coalescing tubercles and necrosis. No significant differences were observed in the gross pathological scores for the lungs, liver and spleen of guinea pigs infected with either strain. Each data point represents the score of an individual animal and the bars depict medians (± interquartile ranges) for each group. (B) The figure depicts representative photomicrographs (20×) of hematoxylin and eosin (H&E) stained 5 µm sections of lung and liver of guinea pigs infected with either wild-type *M.tuberculosis* or MtbΔ*end*Δ*xthA* euthanized at 10 weeks post-infection. Similar histopathological damage was observed in the lung and liver of guinea pigs infected with MtbΔ*end*Δ*xthA* or wild-type *M.tuberculosis*. Infected animals exhibited numerous foci of granulomatous infiltration. The scale bars depict 500 µm.

At 10 weeks post-infection, the guinea pigs infected with MtbWT, MtbΔ*end*, MtbΔ*end*Comp, MtbΔ*xthA*, MtbΔ*xthA*Comp or MtbΔ*end*Δ*xthA* exhibited extensive pathological damage in the lungs, liver and spleen. The pathological damage at this time point was more severe when compared with the damage at 4 weeks post-infection and the organs exhibited heavy involvement with the presence of numerous large tubercles. However, the pathological damage in the animals from all groups was comparable. A comparison of gross pathological damage and gross pathological scores for the organs of animals infected with MtbWTand MtbΔ*end*Δ*xthA* is shown in Fig. 5A. At this time point, the histopathological analysis of lung and liver of the animals infected with MtbWT, MtbΔ*end*, MtbΔ*end*Comp, MtbΔ*xthA*, MtbΔ*xthA*Comp or MtbΔ*end*Δ*xthA* also demonstrated a comparable tissue damage further supporting the gross pathological observations. The lungs of the guinea pigs infected with any of the strains displayed multiple well-defined coalescing granulomas, covering ∼70–80% area of the lung resulting in the loss of lung micro-architecture. Similarly, the animals belonging to different groups exhibited effacement of a large proportion of hepatic parenchyma by multi-focal necrotic granulomas corresponding to ∼30–40% of the liver area. A comparison of histopathological damage to the organs of animals infected with MtbWT and MtbΔ*end*Δ*xthA* is shown in Fig. 5B. A lack of distinction in the histopathological damage to the organs of animals infected with the wild-type *M.tuberculosis* or with the mutant strains shows that the absence of AP endonucleases does not influence the growth of *M.tuberculosis* in guinea pigs.

## Discussion

In the host, *Mycobacterium tuberculosis* is exposed to an environment that is rich in reactive oxygen and nitrogen intermediates capable of damaging the genome of the pathogen [39], [40]. It has been thought that the BER pathway, which repairs the cytotoxic and mutagenic AP sites in DNA, may play a major role in maintaining the integrity of DNA in mycobacteria [26], [41], [42]. However, the experimental evidence gathered by us in this study suggests that though under *in vitro* conditions, the AP endonuclease(s) protect the pathogen against oxidative and alkylation damage, these enzymes are not crucial for the growth and pathogenesis of *M.tuberculosis* in the guinea pig model of infection.

In response to the alkylating agent MMS, AP sites are generated via spontaneous and enzymatic hydrolysis of glycosidic bonds [22]. Our results demonstrate that End and XthA are able to compensate the absence of each other to repair the DNA damaged in response to alkylation stress. However, a simultaneous disruption of both the AP endonucleases in *M.tuberculosis* (MtbΔ*end*Δ*xthA*) significantly reduces the ability of the pathogen to withstand alkylation stress when compared with wild-type *M.tuberculosis*, thereby indicating the importance of AP endonucleases in protecting the pathogen against alkylation damage. In the previous studies, it has been demonstrated that the AP endonuclease mutants of *E.coli* and *B.abortus* exhibit a significantly enhanced sensitivity to MMS when compared with the parental strain, indicating the involvement of AP endonucleases in the repair of MMS induced DNA lesions [22], [25].

H_2_O_2_ generates nicks and breaks in DNA with blocked 3′ termini apart from giving rise to abasic sites [43], [44]. Our results on the role of these AP endonucleases against oxidative damage by employing H_2_O_2_ demonstrate that while both the AP endonucleases make an important contribution in the repair of DNA damage in *M.tuberculosis*, End plays a more important role than XthA in protecting this pathogen against oxidative assault.

The intra- and interstrand cross-linking of DNA resulting from the damage caused by MMC is repaired by nucleotide excision and recombination repair pathways rather than BER in bacteria [45]. However, based on microarray and quantitative RT-PCR analysis Rand *et al.*, reported that addition of MMC to *M.tuberculosis* induces the expression of *xthA* indicating a plausible role of XthA in the repair of DNA damage induced in response to MMC [46]. This tempted us to evaluate the role of these AP endonucleases in the repair of MMC induced DNA damage. However, the experimental evidence gathered in our study does not support any apparent role of XthA or End in the repair of such damage to DNA.

Our studies demonstrate that the disruption of any one of the AP endonucleases in *M.tuberculosis* (MtbΔ*end* or MtbΔ*xthA*) did not affect the growth of the pathogen in THP-1 macrophages when compared with the parental strain. However, disruption of both the AP endonucleases in *M.tuberculosis* (MtbΔ*end*Δ*xthA*) significantly reduced the growth of the pathogen in THP-1 cell line. These observations may be attributable to the inability of *M.tuberculosis* to repair the DNA damage inflicted upon by ROI and RNI produced by the THP-1 macrophages, in the absence of both the AP endonucleases. However, in the guinea pig infection model, we observed that disruption of either one or both the AP endonucleases did not affect the growth of *M.tuberculosis* apparently indicating that these AP endonucleases may not be indispensable for the growth and pathogenesis of *M.tuberculosis*. Our observations were substantiated by gross pathological and histopathological damage.

The difference in the influence of mutations *in vitro* and in the host, we think, may be related to the fact that the autonomous *in vitro* response of the bacterium in the macrophages may be limited. However, *in vivo*, the outcome may depend upon the host-pathogen interaction resulting in the induction of gene expression that is vital for the bacterium to survive in the face of assault mounted by the host. In this context, it is important to note that *M.tuberculosis* has ada operon responsible for what is called the Ada response, which is an adaptive response to alkylation damage [47], [48]. This ada operon encodes a composite protein of AdaA and AlkA and a separate AdaB/Ogt protein [48]. By employing an *M.tuberculosis* mutant lacking the *ada* operon and the parental strain, Durbach *et. al.* demonstrated that the mutant grew normally and was hypersensitive to the alkylation damage *in vitro* but displayed no attenuation *in vivo* in murine model of infection [49]. Such apparent differences in the function of the proteins responsible for Ada response, or AP endonucleases such as End or XthA *in vitro* and *in vivo* in *M.tuberculosis*, may be attributable to either variations in the DNA damage inflicted upon the pathogen during its growth *in vitro* and in the host or due to redundancy in such activities evolutionarily developed by the pathogen to safeguard its genome. The phenotype of MtbΔ*end*Δ*xthA* also resembles the phenotype of a *recA* mutant of *M.bovis* BCG, that has also been demonstrated to exhibit hypersensitivity to DNA damage *in vitro* but displayed no growth impairment in mice model of infection [50]. Besides, there is an increasing evidence of the existence of alternative pathways that cater to damages generally repaired by AP endonucleases and such enzymes are found across different species from bacteria to fungi [51], [52], [53], [54].

The DNA polymerase X of *B.subtilis* (PolX_Bs_), another gram positive organism, has been shown to possess an intrinsic AP endonuclease activity that enables it to recognize, incise, and further repair abasic sites in a AP-endonuclease-independent manner [54]. This persuaded us to search for AP endonuclease analogues in *M.tuberculosis* genome. We found a conserved hypothetical protein in *M.tuberculosis* (Rv3856c) that displayed 39% identity and 57% similarity to the C-terminal polymerase and histidinol phosphatase (PHP) domain of PolX_Bs_. In *B.subtilis*, the AP endonuclease activity of PolX_Bs_ maps at the PHP domain [54]. Multiple sequence alignment of the PHP domain of bacterial/archaeal PolXs has resulted in the identification of highly conserved residues assembling a catalytic core required to coordinate the metal ions important for the activity of this protein [55]. All these residues (H101, H103, H133, E172, H199, H227, E262, D292 and H294) were found to be conserved in the *M.tuberculosis* protein encoded by Rv3856c [56]. Based on these observations, it appears possible that *M.tuberculosis* may also possess repair pathways or proteins that overlap with AP endonucleases in order for it to protect its DNA from damage during its survival in the host.

We demonstrate that *M.tuberculosis* AP endonucleases are important in protecting the pathogen against alkylation and oxidative damage *in vitro* as well as for its growth in human macrophage cell line. However, *M.tuberculosis* may induce overlapping repair pathways and proteins to protect its DNA from damage during its survival in the host that make the role of these AP endonucleases *in vivo* dispensable.
